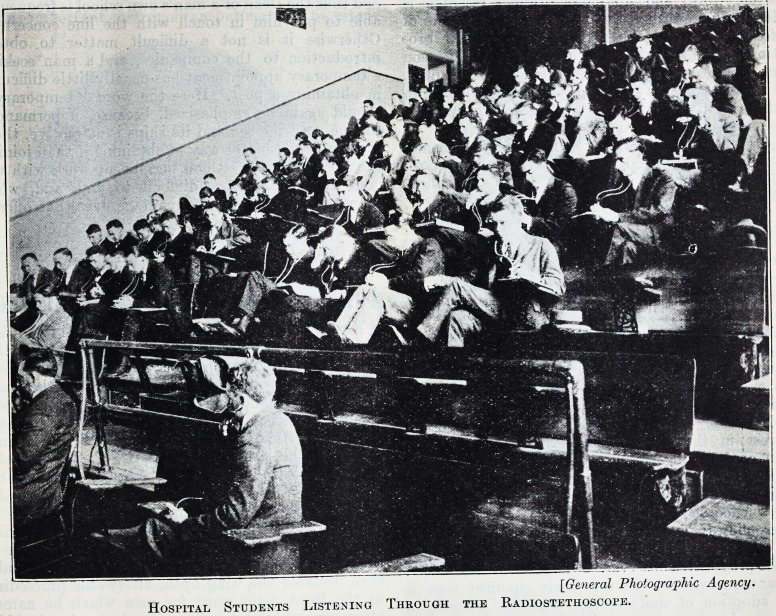# Heartbeats by Radio

**Published:** 1924-09

**Authors:** 


### HEARTBEATS BY RADIO.

### A NEW USE FOR WIRELESS.

The stethophone, or radiostethoscope, has now made it possible for doctors on shore or on board a large liner to listen to the heartbeats of a sick man on some smaller boat that has no medical officer. Diagnosis can then be given and treatment prescribed by wireless. Furthermore, the radio stethoscope has proved capable of detecting murmurs and other important sounds not audible through the ordinary doctor's instrument. Eventually it is probable that phonograph records of typical cases of normal and abnormal heartbeats will be made by this means and used in all medical schools, while physicians will probably be able to make diagnoses by hearing the patient's heartbeat across an ordinary telephone with the aid of the stethophone to amplify the beats.

A Bedside Demonstration. [*General Photographic Agency.*

### The Collective Stethoscope.

A class of more than a hundred medical students has assembled in a clinical auditorium to study diagnosis. A patient is brought into the room, but the students do not file past him, each in turn using his stethoscope on the man's heart. Instead, each of the large audience has an instrument about his head with wires connecting it to the master stethoscope that is pressed over the subject's heart. Each student hears the patient's heartbeat even more distinctly than if he were himself applying the ordinary instrument. Later in the day the students listen in the same way to phonograph records of typical cases of heart trouble. These are some of the possibilities of the radiostethoscope, or stethophone, recently perfected by the engineers of the Western Electric Company after varied experiments had been made for a number of years, notably by Dr. Leo Jacobsohn, and by the United States Army. It makes possible the amplification of the sounds heard through the common medical stethoscope. At the last convention of the American Medical Association, more than five hundred physicians sitting in the theatre on the Municipal Pier, Chicago, heard heartbeats, murmurs, and lung sounds as distinctly as though they had put their own stethoscopes directly on the patient, instead of holding them against telephone receivers. The sounds were picked up by a special device held against the patient by the demonstrating physician. This was the first time that heart murmurs and lung sounds have been transmitted to so large a group of listeners.

### A "Tea Wagon" Amplifier.

The device, which is really a three-stage electrical amplifier, equipped with suitable pick-up and listening apparatus, was the result of long experimentation. The amplifier with its batteries and filters is mounted on a box set on wheels, thus giving the suggestion of a tea wagon. An essential feature of the amplifier, and one in which it differs from other attempts at the use of amplification in auscultation, is its ability to go down to frequencies as low as the human ear can perceive. Hence, through it can be distinguished all sounds that can be heard through the ordinary acoustic stethoscope.

THE SUPPRESSION OF SOUNDS.

A novel and very useful adjunct of the system is the provision for suppressing certain sounds by means of electric filters. These are networks of coils and condensers inserted in the circuits by key switches. It has been found that practically all sounds of interest in auscultation lie below 1,100 cycles per second, and that most lie below 650 cycles. The narrower the range of tones which the listener hears, the more noise is excluded, and, therefore, the better can he concentrate on the really important sounds. With the assistance of several prominent physicians, a group of filters has been selected which will cut off sounds lying above 1,100, 650, 400, and 95 cycles, respectively, and below 130 cycles. For instance, the filter cutting off sounds above 95 cycles is useful in listening to fœtal hearts, and that which cuts off sounds below 130 cycles suppresses the heavy thump of heartbeats, and allows murmurs and chest sounds—usually higher pitched—to be heard more clearly.

HELP IN DIAGNOSIS.

It may be that diagnoses not otherwise possible can be made by the use of the stethophone, for its limits of usefulness have not yet been established. In the examination of thirty-four cases known to have murmurs, the radiostethoscope disclosed additional murmurs in three of these patients. In a case of aneurism, discovered by X-ray examination, several observers could hear a systolic murmur otherwise inaudible. It is highly probable that râles of incipient tuberculosis can be detected earlier by the stethophone—a matter of considerable social importance because the earlier curative measures are taken, the less time will the patient lose from work, and the greater the chance of cure.

BEDSIDE DEMONSTRATIONS.

It is now possible to demonstrate interesting cases where the patient is too ill to stand the strain for the time required by the old method—several hours for a large class. By wheeling the stethophone to the bedside and connecting the receivers by wires, it is possible to demonstrate cases which cannot be brought into the classroom. This is especially true of pneumonia cases in which absolute quiet is so important that students are seldom allowed to listen to the patient's lungs. By using the newly perfected device, an entire class may listen in a distant room at the time of the necessary routine visit of the physician. When, in the midst of a demonstration, the instructor wishes to address the class, he keeps the chest-piece in position against the patient's body and, bringing his lips within several inches of it, talks in a quiet voice. The vibrations are picked up by the patient's body and communicated to the chest-piece, then amplified, and delivered to the students' receivers.

[*General Photographic Agency.*

HOSPITAL STUDENTS LISTENING THROUGH THE RADIOSTETHOSCOPE.

## Figures and Tables

**Figure f1:**
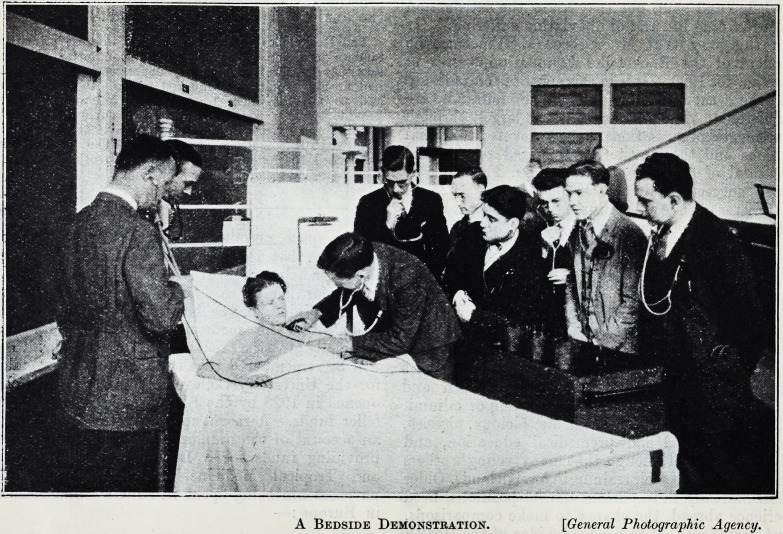


**Figure f2:**